# Market Assessment of Tuberculosis Diagnostics in Brazil in 2012

**DOI:** 10.1371/journal.pone.0104105

**Published:** 2014-08-06

**Authors:** TB Diagnostics Market Analysis Consortium

**Affiliations:** Fundacion Huesped, Argentina

## Abstract

**Background:**

Improved diagnostics for the diagnosis of tuberculosis (TB) are urgently needed. However, test developers and investors require market size data to support new product development. This study assessed the served available market for TB diagnostics in Brazil in 2012 and the market segmentation in the public and private sectors.

**Methods:**

Data were collected on test volumes done in the public and private sectors for the diagnosis of latent and active TB, drug susceptibility testing and treatment follow-up. Tests included were tuberculin skin tests, interferon-gamma releases assays, smear microscopy, solid and liquid cultures, nucleic acid amplification tests and phenotypic drug susceptibility tests. The data were collected by means of an electronic survey via the Brazilian State laboratories and from sales information provided by manufacturers. Test costs for the public sector were calculated using a components approach, while costs for the private sector were based on prices paid by patients. The overall market value (expenditure) for the entire country was calculated using the public sector test costs.

**Results:**

During 2012, an estimated total of 2.4 million TB diagnostic tests were done in Brazil, resulting in an estimated overall market value of USD 17.2 million. The public sector accounted for 91% of the test volumes and 88% of the market value. Smear microscopy was the most commonly test (n = 1.3 million; 55% of total) at an estimated value of USD 3.7 million. Culture overall (n = 302,761) represented 13% of test volumes and 40% (USD 6.9 million) of the market value. On average, USD 208 was spent on TB diagnostics for every notified TB patient in Brazil, in 2012.

**Conclusion:**

The TB diagnostics market value in Brazil in 2012 was over USD 17 million. These study results will help test developers to understand the current and potential market for replacement or add-on diagnostic technologies.

## Background

In 2012, despite a very gradual decline in the number of new tuberculosis (TB) cases globally, it was estimated that 8.6 million people developed TB and 1.3 million patients died [1]. Drug-resistant TB, including multi-drug resistant TB (MDR-TB), has been found worldwide, though the actual numbers of cases are not accurately known due to poor rates of detection. Furthermore, the burden of TB cases is thought to be significantly under-estimated due to the approximately 34% or 3 million TB cases that are never diagnosed or are not reported [1].

Appropriate, quality tests for the diagnosis of TB and the detection of drug resistance are critical for TB programmes and for optimal patient outcomes. There have been a number of advances in TB diagnostic technologies since 2006 which have received endorsement from the World Health Organization (WHO), including fluorescence microscopy, liquid–based culture systems and nucleic acid amplification tests (NAAT) such as the Xpert MTB/RIF (Cepheid Inc. Sunnyvale, California, U.S.). These tests are now being introduced in many countries [2], [3]. However, there still remains a great need for other tests, such as a true point of care (POC) test which can be used at all levels of the health care system [4] and could potentially enable treatment initiation during the patient’s first medical visit. The UNITAID TB diagnostic landscape report reviews currently available tests as well as those in the development process or at early market entry [5].

An understanding of the size, value and dynamics of the current and potential market for new diagnostics is critical for decisions regarding the research and development needed in order to bring new tests to market, and to predict whether they will generate a return on investment [6]. The first and only global market assessment for TB diagnostics was published in 2006 by the Foundation for Innovative New Diagnostics (FIND) and The Special Programme for Research and Training in Tropical Diseases (TDR). The report showed that over 1 billion dollars (USD) was spent worldwide on TB diagnostics [7]. The diagnostic landscape has changed a great deal since then and test manufacturers need current market information at the national level, particularly in countries with emerging economies that also have a high burden of TB, such as the BRICS (Brazil, Russia, India, China, South Africa) countries [6]. While it is difficult to predict the total potential available market, where all possible TB suspects would be tested, a more realistic market indicator is the served available market (SAM), which is based on the actual number, cost and type of tests in current use. This study assessed the SAM for TB diagnostics in Brazil in 2012. The volume and cost of the various tests that were in use for the diagnosis of latent and active TB, drug susceptibility testing (DST) and treatment follow-up, as well as their market segmentation in the public and private sectors and the overall market values, are described.

## Material and Methods

### Setting

Brazil, a middle income country with a total estimated population of 199 million in 2012 [8] had an estimated TB incidence rate of 46 per 100.000 population and 82,755 reported cases; 71,230 of which were new cases. The estimated burden of MDR-TB amongst new cases was 1.4% and was 7.5% for re-treatment cases [1]. There is a national network of public health TB laboratories in Brazil that work with the National TB Control Program (PNCT), under the guidance of the Coordination of Public Health Laboratories (CGLAB) and the National Reference Laboratory (NRL). There is a central public health laboratory (LACEN) in each of the 26 states and one in the Federal District, as well as approximately 4000 local public and private laboratories that provide TB testing [9]. Many of the local laboratories report data to the 27 LACENs. In addition, there are collaborating centers such as universities and hospitals that carry out research activities as well as some diagnostic services. Due to the high expense for laboratory services, many states and municipalities outsource laboratory testing to private laboratories. Likewise, private laboratories may send part of their testing work, such as DST, to public laboratories.

### Testing algorithsm

The algorithm for bacteriological TB testing in Brazil in 2012 [10] was as follows; two smears were recommended for all persons with respiratory symptoms or patients with radiological or clinical suspicion of TB. Culture was indicated if persons with presumptive TB were smear negative, or if extrapulmonary TB was suspected. Culture plus DST was indicated for new cases with a known contact of drug-resistant TB, for high-risk new cases such as health care workers, indigenous and homeless people etc., and for patients with a positive smear after 2 months of treatment or with treatment failure.

### Tests included in this market analysis

Data on the volume of tests done and individual test costs, in the public and private sectors, were collected for the following diagnostics used for the detection of latent TB (LTBI), for the initial diagnosis of active TB and for follow-up testing: tuberculin skin tests (TST), sputum smear microscopy (SSM), culture, NAATs such as the Xpert MTB/RIF, and first and second-line phenotypic DST. Interferon-gamma release assays (IGRA) and line probe assays (LPA) were not in routine use in Brazil in 2012, and complete data on chest x-rays, the adenosine deaminase test, blood cultures, speciation tests other than molecular and histopathology were not available, therefore they were excluded.

### Calculation of test volumes in Brazil

Due to differences in data availability, three methods were used for the collection of data on test volumes in this study. A bottom-up approach was used by means of an electronic survey which was circulated to each of the LACENs for the collection of TB diagnostic testing data for their states. Where available, the LACENs provided data for all laboratories within their state networks, which in some cases also included local private laboratories. This bottom-up approach was used for the collection of test volumes in the public sector for solid and liquid media culture, PCR and first and second-line DST and in the private sector for solid media culture. When accurate test volume data were not available from the survey, a top-down approach was used. Information on reagent volumes for commercial tests sold in Brazil in 2012, were obtained from manufacturers and used for test volume calculations (e.g. TST, Xpert MTB/RIF). Thirdly, a middle-out approach was used where some primary on-site data collection was required; for instance to calculate reagent use per test, frequency of test repeats and quality controls (e.g. use of liquid media culture in the private sector). No ethical approval was required for the study, since only aggregated test volume data was collected by the LACENs and no data was collected on an individual patient level.

Though the LACENs were requested to complete the survey with test volumes for their entire state, in some cases this information was limited due to the significant number of local laboratories that did not report to the state laboratories. It was suggested that these would usually be small peripheral laboratories that most likely performed only SSM, therefore the total number of smears done nationally was estimated. Nine states reported complete data for all their laboratories for the number of diagnostic smears done. These smear volumes (diagnostic smears only) were divided by the assumed average number of smears done per investigation (1.5) to determine the number of TB suspects tested in these states. Subsequently, the number of TB supsects were divided by the number of new pulmonary TB (PTB) cases diagnosed in each of these states, which resulted in an average case to suspect ratio of 1∶15. This ratio was then applied to the number of new PTB cases for the entire country in 2012 in order to estimate the smear volume for the whole country. In addition, the two smears recommended for follow-up and for re-treatment cases were added, to give a final estimate of the total volume of smears done nationally in 2012. For the nine states that had accurate information on the number of smears done in both the public and private sectors, the proportion of smears done in the private sector was calculated. This proportion of smears was considered to be representative of the private and public sector distribution in the entire country, since the proportion of public versus private labs across the states was relatively constant (average 62% of all labs within each state were public labs versus 38% private labs) and was used to divide the total number of smears done nationally into the two sectors.

The volume of TSTs was estimated via a top-down approach since TST is usually done in hospitals or by the PNCT in screening programmes, rather than in laboratories. The number of TST vials sold in Brazil was provided by the single distributor for purified protein derivative (PPD) in the country. An average number of 10 tests per vial (of 1.5 ml) based on expert opinion, was used to estimate the total number of TSTs done. Likewise, a top-down approach was used for Xpert MTB/RIF, using the number of cartridges sold in the country in 2012 as the volume of tests done, for the public and private sectors. Since complete data on the number of liquid media cultures and DST were not available for the private sector from the survey, these were calculated by a top-down and middle-out approach, as was the number of pyrazinamide (PZA-DST tests in liquid media), for each sector.

### Calculation of test costs in Brazil

All test costs in this report are in USD and prices are for 2012. Details of test components and costs are described below. The World Bank official exchange rate for 2012 was used to convert Brazilian Reais into USD (exchange rate was 1∶0.513).

#### Public sector

Test costs were estimated by the project team, based on information gathered during visits to two LACEN laboratories and the central laboratory in São Paulo. It should be noted that a detailed costing exercise was not the purpose of this study. A standard Excel template was used to compile unit costs for each diagnostic test for reagents and consumables, labour and equipment. For laboratory overheads (electricity, water, etc.), an overall rate of 7% was applied. Capital costs were annualised using the standard discount rate of 3% [11]. For Xpert MTB/RIF the cost was calculated by the PNCT during a pilot implementation study in two cities.

#### Private sector

It was not possible to access the actual test costs in the private sector, however several members of our consortium, were able to gather the prices charged to the patient from eight main private laboratories, for a variety of tests. This information was used to calculate an average price charged per test. In the absence of an actual cost, these prices were used as the test cost for the private sector.

#### Overall market value (both sectors)

To estimate the overall value of the TB diagnostic market in Brazil in 2012, including the public and private sectors, the public sector cost per test was used since these costs were thought to best reflect the actual costs for conducting the tests, without incorporating any markup.

### Calculation of the value of the 2012 served available market in Brazil, by public versus private segmentation

Three different market values were calculated. First, the value of the public-sector market was calculated by multiplying the volume of each of the TB diagnostic tests in the public sector by the public-sector test cost. Secondly, the value of the private-sector market was calculated by multiplying private-sector test volumes by the average private-sector price charged to the patient. Lastly, the value of the overall TB diagnostic market was calculated by multiplying the total number of each test done in both sectors by the public sector cost, as defined above.

### Sensitivity analysis

An average monthly salary of 3000 Reais was used for all test cost calculations in the primary analysis.

A one-way sensitivity analysis was undertaken because salary scales for technical staff are known to differ widely across different regions in Brazil and may also depend on the level of technical staff who perform the test. Salaries were reported to range from 1000 to 6000 Brazilian Reais per month, and this range was applied to the costs of TST, SSM, culture and DST in the sensitivity analysis. Costs for consumables, equipment and overheads were considered to remain constant.

To account for uncertainty in the true smear volume that we estimated, we report an uncertainty range. The lower limit represents the smear volume as reported in the survey, knowing that this might be an under estimation. The upper limit is the number of smears as reported in the Brazilian Ministry of Health database (DATASUS), which registers all clinical investigations done for reimbursement purposes [12].

## Results

### Public sector

The 27 LACEN public laboratories provided SSM, culture and first-line DST, with 13 laboratories using liquid media and 14 laboratories using solid media (Lowenstein-Jensen (LJ), or Ogawa). The local laboratories provided a variety of services from SSM only, to a few with culture and DST capabilities. Information for a total of 1,073 public laboratories and 1,710 private laboratories was reported in the survey, including all LACEN laboratories, though information was lacking for a number of peripheral microscopy laboratories. All laboratories used the Ziehl-Neelsen (ZN) staining method for SSM. For cultures done in the public sector, 35% used liquid media and 65% solid media. For first-line DST, either liquid media (63%) or LJ media (37%) was used and the first-line panel generally consisted of 4 drugs, streptomycin, isoniazid, rifampin and ethambutol, (SIRE), with some laboratories that used liquid media also testing PZA. The NRL and the central laboratory in São Paulo provided second-line DST and the latter used PCR for speciation and for the detection of TB in spinal fluid samples.

The total volume of tests done in the public sector amounted to 2,158,664 tests at a market value of USD 15,107,921 (Table 1), representing 17.6% of the estimated NTP budget in Brazil for that year [13]. The largest volume of tests was SSM, which represented 53% of all tests and 22% (USD 3.2 million) of the total market value for this sector (Figures S1 and S2 in File S1). The largest contributor to the market value was culture, which represented 12% of the test volumes but 37% (USD 5.6 million) of the total market value. TST was also a significant contributor to this market, representing 33% of test volumes and 28% (USD 4.3 million) of the overall value in the public sector. During eight months of 2012, a pilot study of Xpert MTB/RIF was undertaken for the diagnosis of PTB in two high-incidence municipalities in Brazil; Rio de Janeiro and Manaus. Some laboratories continued Xpert MTB/RIF use after the study period, amounting to an overall total of 18,000 tests done. The breakdown of costs by different components for tests done in the public sector is shown in Table 2.

**Table 1:** Total costs of TB diagnostic tests and market value in the public sector in Brazil, 2012, in USD.

Diagnostic test	Cost pertest	No. of testsdone inpublic sector	% of totalvolume,public sector	Total value,public sector	% of total value,public sector
TST	5.90	725,000	33.6%	4,278,555	28.3%
IGRA	Not done	Not done			
Sputum smearmicroscopy (ZN)	2.85	1,139,367	52.8%	3,245,596	21.5%
Culture (liquid)	31.43	91,445	4.2%	2,874,409	19.0%
Culture (solid)	16.36	168,432	7.8%	2,756,024	18.2%
Xpert MTB/RIF	17.80	18,000	0.8%	320,400	2.1%
LPA	Not done	Not done			
PCR	44.99	614	<0.1%	USD 27,624	0.2%
First line DST(liquid, SIRE)	130.54	9,049	0.4%	USD 1,181,212	7.8%
First line DST(liquid, SIREP)	196.65	539	<0.1%	USD 105,995	0.7%
First line DST(solid, SIRE)	41.11	5,519	0.3%	226,861	1.5%
Second line DST(liquid, 4 drugs)	130.54	699	<0.1%	91,244	0.6%
**Total**		**2,158,664**	**100%**	**15,107,921**	**100%**

TST = tuberculin skin test; IGRA = Interferon gamma release assay; ZN = Ziehl-Neelsen; LPA = line probe assay; PCR = polymerase chain reaction; DST = drug susceptibility test; SIRE = Streptomycin, Isoniazid, Rifampin, Ethambutol; SIREP = Streptomycin, Isoniazid, Rifampin, Ethambutol, Pyrazinamide; USD = United States Dollars.

**Table 2:** Calculated costs for TB diagnostic tests for the public sector, breakdown by components.

	TST	sputum smearmicroscopy(ZN)	culture(liquid)	culture(solid)	XpertMTB/RIF#	PCR‡	First lineDST(liquid, SIRE)	First lineDST(liquid, SIREP)	First lineDST(solid, SIRE)
**Consumables**	$2.31	$0.32	$26.67	$10.26		‡	$117.02	$178,38	$31.28
**Instrument utility**	N/A	$0.41	$0.57	$0.88			$1.56	$1.56	$0.72
**Labor**	$3.21	$1.92	$2.14	$3.74			$3.42	$3.85	$6.41
**Overhead (non-reportable** **results, repeats,** **sample transport** **and lab operating expenses)**	$0.39	$0.19	$2.06	$1.04			$8.54	$12.87	$2.69
**Total (USD)**	**$5.90**	**$2.85**	**$31.43**	**$16.36**	**$17.80**	**$44.99**	**$130.54**	**$196.65**	**$41.11**

TST = tuberculin skin test; ZN = Ziehl-Neelsen; PCR = polymerase chain reaction; DST = drug susceptibility test; SIRE = Streptomycin, Isoniazid, Rifampin, Ethambutol; SIREP = Streptomycin, Isoniazid, Rifampin, Ethambuthol, Pyrazinamid; USD = United States Dollars.

# Costing is based on Xpert implementation study.

‡ Costing done by one of the laboratories (no breakdown known).

### Private sector

It was estimated that the private sector performed a total of 225,199 TB diagnostic tests (Table 3, Figures S1 and S2 in File S1) in 2012.

**Table 3:** Total prices for TB diagnostic tests in the private sector and market value in Brazil in 2012, in USD.

Diagnostic test	Price chargedfor test	No. of tests donein private sector	% of total volume,private sector	Total value, privatesector	% of total value, privatesector
TST	No info	No info			
IGRA	Not done	Not done			
Sputum smearmicroscopy (ZN)	18.97	176,482	78.4%	3,348,633	47.6%
Culture (liquid)#	50.77	37,124	16.5%	1,884,755	26.8%
Culture (solid)#	50.77	5,760	2.6%	292,430	4.2%
Xpert MTB/RIF	254.77	3,400	1.5%	866,215	12.3%
LPA	Not done	Not done			
PCR	Not info	No info			
First line DST(liquid, SIRE)	263.84	2,433	1.1%	641,927	9.1%
First line DST(liquid, SIREP)	No info	No info			
First line DST(solid, SIRE)	No info	No info			
Second line DST(liquid, 4 drugs)	No info	No info			
**Total**		**225,199**	**100%**	**7,033,960**	**100%**

TST = tuberculin skin test; IGRA = Interferon gamma release assay; ZN = Ziehl-Neelsen; LPA = line probe assay; PCR = polymerase chain reaction; DST = drug susceptibility test; SIRE = Streptomycin, Isoniazid, Rifampin, Ethambutol; SIREP = Streptomycin, Isoniazid, Rifampin, Ethambuthol, Pyrazinamid; USD = United States Dollars.

Note: Private sector market value estimate using the price charged to the patient per test, as the test cost.

# No distinction could be made between prices charged for liquid or solid culture by private laboratories; therefore all the same value was used for liquid and solid culture.

Using the price charged to the patient, the overall private market amounted to USD 7 million, while if the public sector test cost was used, the private market estimate was USD 2 million. Private laboratories did not have access to FIND-negotiated reduced pricing for reagents, as did the public sector, therefore their costs for liquid culture and Xpert MTB/RIF would have been higher than in the public sector. Prices charged by private sector laboratories also reflect, in part, their operating profit margins and were on average 5.5 times higher than the unit test costs estimated for the public sector, though the comparison of prices with costs may not be reasonable.

The most commonly used test was SSM with 176,482 tests representing 78% of all tests done in the private sector. The value for SSM was USD 3.3 million using the price charged (or USD 0.5 million using the public sector test cost). For culture, 42,884 tests were done (19% of all tests), with a value of USD 2.2 million using the price charged to patients (or USD 1.3 million using the public sector cost). Overall the private sector contributed 9.4% of the national volume of tests done and 12% of the market value when using public sector costs. In the private laboratories use of liquid media was more common for culture and DST than in the public sector. Liquid media was used for 87% of all cultures and 100% for DST in private laboratories.

### Overall market and market segmentation

For the overall market, the price per test estimated for the public sector was applied to all tests, including those done in the private sector. The total number of TB diagnostic tests done in Brazil in 2012 was 2.4 million, with a market value of USD 17.2 million (Table 4, Figures S1 and S2 in File S1). Of these tests, 91% were done in the public sector.

**Table 4:** Total value of the overall TB diagnostic market, public and private sectors combined, using public sector costs, in Brazil in 2012, in USD.

Diagnostic test	No. inpublicsector	% publicsector	No. inprivatesector	% privatesector	Total no.tests done	% of totalvolume	Cost pertest	Overall marketvalue	% of totalvalue	Sensitivityanalysis:lower-upperrange
TST	725,000	100%	No info	0%	725,000	30.4%	5.90	4,278,555	24.8%	2,729,412–6,602,271
IGRA	Not done		Not done				Not done			
Sputum smearmicroscopy (ZN)^1^	1,139,367	86.6%	176,482	13.4%	1,315,849 (range: 900,954–1,409,736)	55.2% (range:45.8%–56.9%)	2.85	3,748,322(range: 2,566,454–4,015,769)	21.7% (range:16.0%–22.9%)	2,061,338–6,278,799(range: 1,411,386–6,726,797)
Culture (liquid)	91,445	71.1%	37,124	28.9%	128,569	5.4%	31.43	4,041,335	23.4%	3,858,189 4,316,055
Culture (solid)	168,432	96.7%	5,760	3.3%	174,192	7.3%	16.36	2,850,274	16.5%	2,338,301–3,423,898
Xpert MTB/RIF	18,000	84.1%	3,400	15.9%	21,400	0.9%	17.80	380,920	2,2%	380,920 (unchanged)
LPA	Not done		Not done				Not done			
PCR	614	100%	No info	0%	614	<0.1%	44.99	27,624	0.2%	27,624 (unchanged)
First line DST(liquid, SIRE)	9049	80.1%	2,433	19.9%	11,482	0.5%	130.54	1,498,804	8.7%	1,472,632–1,538,059
First line DST(liquid, SIREP)	539	100%	No info	0%	539	<0.1%	196.65	105,995	0.6%	104,613–108,068
First line DST(solid, SIRE)	5,519	100%	No info	0%	5,519	0.2%	41.11	226,861	1.3%	203,275–262,239
Second line DST(liquid, 4 drugs)	699	100%	No info	0%	699	<0.1%	130.54	91,244	0.5%	89,651–93,634
**Total**	**2,158,664**	**90.6%**	**225,199**	**9.4%**	**2,383,863** **(range: 1,968,968–** **2,477,750)**	**100%**		**17,249,936** **(range: 16,068,067–** **17,517,382)**	**100%**	**13,265,957–23,032,567** **(range: 12,616,005–** **23,479,565)**

TST = tuberculin skin test; IGRA = Interferon gamma release assay; ZN = Ziehl-Neelsen; LPA = line probe assay; PCR = polymerase chain reaction; DST = drug susceptibility test; SIRE = Streptomycin, Isoniazid, Rifampin, Ethambutol; SIREP = Streptomycin, Isoniazid, Rifampin, Ethambuthol, Pyrazinamid; USD = United States Dollars.

1 Range in sputum smear microcopy volume and corresponding market value show the uncertainty range around the true number of smears done in the country. The lower estimate is the total number of smears reported as reported by the States in the survey. The upper estimate is the total number of smears reported in the Brazilian Ministry of Health database (DATASUS).

When the public and private sectors were combined, the most common test in 2012 was still SSM (n = 1.3 million), representing 55% of all tests and 22% (USD 3.7 million) of the market value. Culture overall (n = 302,761) represented 13% of test volumes and 40% (USD 6.9 million) of the market value. Culture-based DST (n = 18,239) contributed 0.8% of the total test volume but 11% (USD 1.9 million) of the market value. The most expensive single test was first-line liquid culture DST for 5 drugs, at an estimated cost per test of USD 196.65. The use of Xpert MTB/RIF was limited to the 8 month pilot study in the public sector and was offered in few private laboratories. The total number of Xpert MTB/RIF tests was 21,400, representing 0.9% of all tests by volume and 2.2% of the market value.

**Figure 1:**
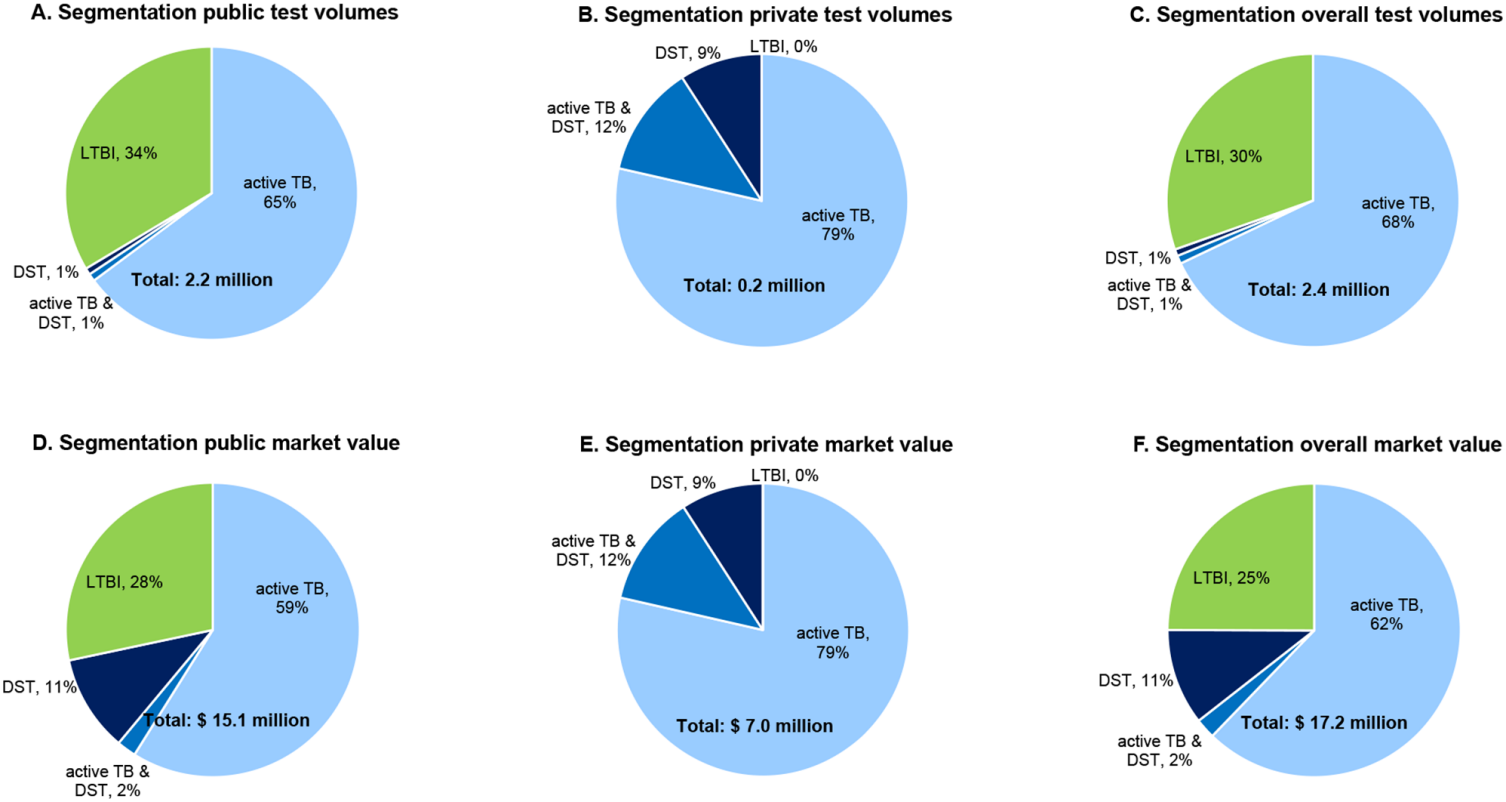
Served available market (volumes and costs), segmented by testing category and health sector. Individual tests were categorized into tests that detect LTBI (tuberculin skin test), those that detect active TB (sputum smear microscopy, liquid and solid culture and polymerase chain reaction tests), those that detect active TB as well as drug resistance (Xpert MTB/RIF), those that solely detect drug resistance once the presence of active TB is already demonstrated (phenotypic liquid and solid media DST). Test volumes and market values are given respectively for the public sector (panels A and D), the private sector (panels B and E) and the public and private sectors combined (panels C and F). Percentages were rounded to the nearest integer. LTBI = latent tuberculosis infection: TB = tuberculosis; DST = drug susceptibility tests.

Tests used for detection of active TB and treatment monitoring (SSM, culture, Xpert MTB/RIF, PCR) represented 68% of the total volume of tests done and 62% of the market value in 2012, while TST, used for latent TB, accounted for 30% of test volumes and 25% (USD 4.3 million) of the total market value (Figure 1). Only 1% of the overall test volume, but 11% of the market value consisted of tests that were solely done for DST, once TB was already diagnosed.

Results of the sensitivity analysis are shown in Table 4. Varying the salaries by a range when calculating the cost of individual tests as well as accounting for uncertainties in the volume of smears resulted in a 27% lower or 36% higher estimation of the overall TB diagnostic market (ranging from USD 12.6 to USD 23.5 million).

## Discussion

This study provides a snapshot of TB diagnostics use in Brazil for the year 2012, including a trial use of the Xpert MTB/RIF test in two high incidence areas of the country. The diagnostics market was estimated in both the pubic and private sectors as well as overall for the country, while recognizing that this is a transitional phase before the planned widespread implementation of the Xpert MTB/RIF in 2014.

Since the previous market analysis conducted by FIND and TDR (which used data from 2003–04), SSM volumes in Brazil seem to have increased considerably by 2012, while the TB incidence rate decreasedPreviously, a total of 0.85 million smears were estimated to have been done, compared to 1.3 million in the current analysis. The number of cultures done annually has remained unchanged since the previous market analysis totalling 0.3 million tests. Our results suggest that for TB diagnostics the private sector is relatively small, although this might have increased slightly in the last decade. In the FIND report, 8% of smears and 4% of cultures were thought to be done in the private sector, while currently these percentages had increased to 13% and 14% respectively. Nevertheles, it cannot be completely ruled out that some of these differences are attributable to slight differences in the methodology and approaches used in the current and past analysis.

One limitation of our analysis is that it covered the period (2012) before the PNCT decision to introduce Xpert MTB/RIF on a larger scale. Our analysis should therefore be interpreted with caution when making inferences on the potential available market. Testing algorithms have already chanced since the introduction of the Xpert MTB/RIF in municipalities with the largest case loads, and the number of individuals that get tested and patients that are diagnosed may change as a consequence. In 2014, Brazil began the roll-out of Xpert MTB/RIF test, and the Brazilian PNCT has procured over 464,000 Xpert MTB/RIF cartridges and 160 instruments, in order to provide the test in 92 municipalities where 50% of all TB diagnoses occurred in 2012. The scale up of Xpert MTB/RIF in the country will likely substantially change the market in the near future, and result in an anticipated significant drop in the number of SSM performed and an increase in testing by Xpert MTB/RIF. Therefore, a future update of this analysis will be helpful to capture such trends. As training of healthcare professionals is undertaken and public laboratories increase their capacity to perform culture for patients with suspected rifampin-resistance, screened by Xpert MTB/RIF, better adherence to recommended culture and DST performance is expected.

There were other limitations in the study; lack of information for the complete private sector and incomplete data for some peripheral laboratories in the public sector may have led to under- estimations of test volumes. The cost per test was estimated using data from a small number of public laboratories in two states, and did not account for fluctuations in costs that may occur in different regions of the country, although a sensitivity analysis was done in an attempt to address this issue. The SSM estimation excluded smears that were done for extrapulmonary TB. The total diagnostic market for TB may in fact be higher than estimated here, because some tests (e.g. chest-X-rays) were excluded since these can be done for the diagnosis of different diseases. Novel TB diagnostics might therefore potentially have a market that is larger than outlined here, when they can replace one or more of the current TB tests, and their technological characteristics allow for implementation in a wider variety of settings and levels of the health care system.

In conclusion, this study showed that the served available market for TB diagnostics in Brazil in 2012 was considerable, with 2.4 million tests done and a market value of over USD 17 million. This corresponds to an average of USD 208 being spent on TB diagnostics per notified TB case. It is recognized that the market is likely to change with the introduction of new technologies and revised algorithms for diagnostic testing. The results of this study will help test developers to understand the potential market for new replacement or add-on technologies and their possible applications in this middle-income, high TB burden country.

## Supporting Information

File S1 **This file includes Figure S1 and S2.**
(DOCX)
