# Correction: Market Assessment of Tuberculosis Diagnostics in Brazil in 2012

**DOI:** 10.1371/journal.pone.0107651

**Published:** 2014-09-05

**Authors:** The PLOS ONE Staff

The email address for the corresponding author is incorrect in the online version of the article. The publisher apologizes for the error. The correct email address is: sandra.kik@mail.mcgill.ca
