# Supplementary material for: Market Assessment of Tuberculosis Diagnostics in Brazil in 2012
Source: PLoS One. 2014 Aug 6;9(8):e104105. doi: 10.1371/journal.pone.0104105 (PMC4123933; doi:10.1371/journal.pone.0104105)

## **Supporting information**

[Figure S1]

**Figure S1. Breakdown of TB diagnostics done in the public and private sector and both sectors combined.**

TB diagnostics are broken down based on the volume of tests done in the public sector (panel A), the private sector (panel B) and public and private sectors combined (panel C), in 2012 in Brazil.

Percentages were rounded to the nearest integer.

TB=tuberculosis; TST= tuberculin skin test; Microscopy= sputum smear microscopy; Xpert= Xpert MTB/RIF; PCR=polymerase chain reaction test; DST=drug susceptibility tests


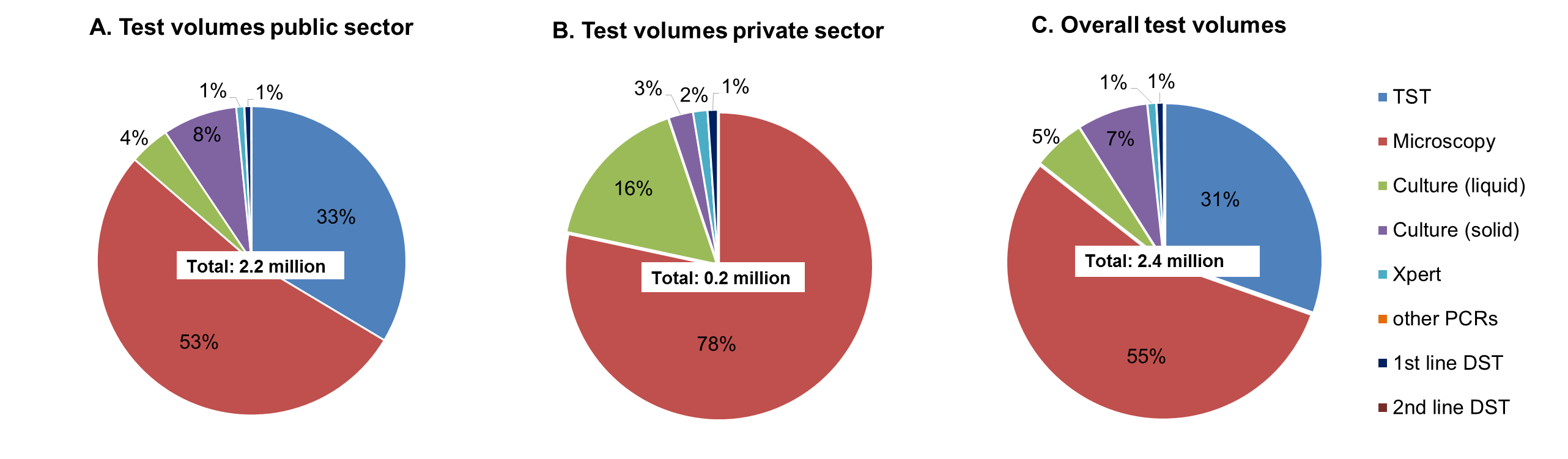


[Figure S2]

**Figure S2. Total costs of TB diagnostics in the public and private sector, and both sectors combined.**

Total costs of TB diagnostics in the public sector (panel A), the private sector (panel B) and the public and private sectors combined (panel C) in Brazil for 2012, are shown. Breakdown by test types.

Percentages were rounded to the nearest integer.

TB=tuberculosis; TST= tuberculin skin test; Microscopy= sputum smear microscopy; Xpert= Xpert MTB/RIF; PCR=polymerase chain reaction test; DST=drug susceptibility tests


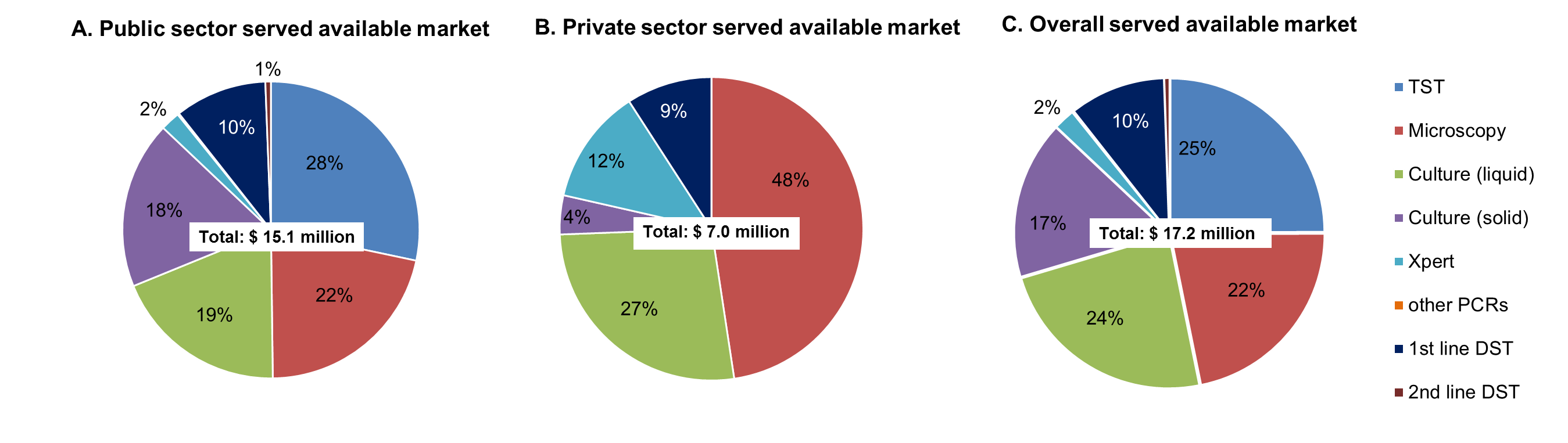

Supplement: File S1 — This file includes Figure S1 and S2. (DOCX) [file pone.0104105.s001.docx]
